# Intestinal serotonergic system is modulated by Toll-like receptor 9

**DOI:** 10.1007/s13105-022-00897-2

**Published:** 2022-06-07

**Authors:** Elena Layunta, Eva Latorre, Laura Grasa, María Pilar Arruebo, Berta Buey, Ana I. Alcalde, José E. Mesonero

**Affiliations:** 1 Institute of Biomedicine, Department of Medical Biochemistry and Cell Biology, University of Gothenburg, Gothenburg, Sweden; 2 Instituto de Investigación Sanitaria de Aragón (IIS Aragón), Zaragoza, Spain; 3 Instituto Agroalimentario de Aragón – IA2- (Universidad de Zaragoza – CITA), Zaragoza, Spain; 4 Departamento de Bioquímica y Biología Molecular y Celular, Facultad de Ciencias, Universidad de Zaragoza, Pedro Cerbuna 12, 50009 Zaragoza, Spain; 5 Departamento de Farmacología, Fisiología y Medicina Legal y Forense, Facultad de Veterinaria, Universidad de Zaragoza, Zaragoza, Spain

**Keywords:** Intestine, Microbiota, PRRs, Serotonin, SERT, TPH

## Abstract

Intestinal serotonergic system is a key modulator of intestinal homeostasis; however, its regulation is still unclear. Toll-like receptor 9 (TLR9), an innate immune receptor, detects different external agents in the intestine, preserving intestinal integrity. Since little is known about TLR9 role in the intestine, our aim was to address the potential regulation between TLR9 and intestinal serotonergic system. Caco-2/TC7 cell line and intestinal tract of *Tlr9*^−/−^ mice were used in this study. Serotonin uptake studies were performed, and molecular expression of different serotonergic components was analyzed by western blot and real-time PCR. Our results show that TLR9 activation inhibits serotonin transporter activity and expression, involving p38/MAPK and ERK/MAPK intracellular pathways, and reciprocally, serotonin increases TLR9 expression. Supporting this interaction, serotonin transporter, serotonin receptors and serotonin producer enzymes were found altered in intestinal tract of *Tlr9*^*−/−*^ mice. We conclude that TLR9 could contribute to intestinal homeostasis by modulation of intestinal serotonergic system.

## Introduction

Serotonin (5-hydroxytryptamine; 5-HT), the main effector of serotonergic system, is chiefly synthesized and released in the intestine. 5-HT is mainly produced by tryptophan hydroxylase 1 (TPH1) in enterochromaffin cells found in the intestinal epithelium, but also in neurons of enteric nervous system by the enzyme TPH2. Extracellular 5-HT availability is modulated by the serotonin transporter (SERT), which is expressed in enterocytes and transports 5-HT into the cells, limiting serotonergic effects. The serotonergic signaling is due to binding of 5-HT to specific receptors, which are widely distributed in all tissues, especially in intestine. 5-HT is widely involved in the regulation of processes associated with intestinal homeostasis such as digestion, secretion, absorption and motility and even intestinal immune function [1].

Within this context, the intestinal innate immune system is indispensable, since the intestinal epithelium acts as first line of defense developing either protective or tolerogenic responses to pathogenic or commensal microorganisms, respectively. Innate receptors such as Toll-like receptors (TLRs) detect and recognize microbe-associated molecular patterns (MAMPs) from bacteria, viruses and fungi [2].

Toll-like receptor 9 (TLR9) is a transmembrane glycoprotein with a LRR domain that recognizes MAMPs and a TIR domain responsible of the downstream signaling after TLR9 activation. This receptor is present in immune cell-rich tissues where several immune cells, such as dendritic cells and B-cells, express high levels of TLR9. In addition, TLR9 is also expressed in non-immune cells including intestinal epithelial cells [3]. TLR9 detects a sequence motif of DNA which consists of a hexamer with central unmethylated CpG. These DNA motifs are abundant in bacteria but are unusual in eukaryotic cells, which allows host through TLR9 differentiates self from foreign DNA [4].

Traditionally, TLR9 has been reported as an intracellular TLR, located in endosomal membranes. However, recent works have indicated that TLR9 could also be expressed in both the basolateral and apical cell membrane where its function seems to be different from endosomal location [4].

Recently it has been reported that activation of some TLRs affect the expression and activity of SERT, describing a system where microbiota could modulate intestinal physiology [1]. However, the involvement of TLR9 in intestinal serotonergic system is unknown. Therefore, the aim of this work has been to study the effects of TLR9 on the intestinal serotonergic system. For this purpose, modulation of SERT activity and expression due to TLR9 activation was analyzed in human intestinal epithelial cells, and expression of different components of serotonergic system (SERT, TPH1, TPH2 and 5-HT receptors) was assessed in ileum and colon of TLR9 knockout mice (*tlr9*^*−/−*^).

## Material and methods

### Reagents and antibodies

The following drugs and reagents were used (abbreviations and respective suppliers in parentheses): serotonin (5-hydroxytryptamine, 5-HT) from Sigma-Aldrich (St. Louis, MO, USA) and [^3^H]-5-HT (specific activity 28 Ci/mmol) from PerkinElmer (Boston, MA, USA). Class C CpG oligonucleotide (ODN2395, ODN), and Pepinh-MYD (MyD88 inhibitor) were acquired from InvivoGen (San Diego, CA, USA). Anthra [1–9-cd]pyrazol-6(2H)-one (SP600125), 1,4-diamino-2,3-dicyano-1,4-bis(2 aminophenylthio)butadiene (U0126) and 4-[5-(4-Fluorophenyl)-2-[4-(methylsulphonyl)phenyl]-1H-imidazol-4-yl]pyridine hydrochloride (SB203580) were acquired from Tocris (Madrid, Spain). Primary antibodies used were: goat polyclonal antibody anti-human/mouse SERT (ab130130) and mouse monoclonal antibody anti-human and anti-mouse [26C593.2] to TLR9 (ab134368) were from Abcam (Cambridge, UK). Goat polyclonal anti-human/mouse actin antibody (SC-1615) and secondary antibodies coupled to horseradish peroxidase were from Santa Cruz Biotechnology (Santa Cruz, CA, USA). All generic reagents, as well as N4-(7-chloro-4-quinolinyl)-N1,N1-dimethyl-1,4-pentanediamine diphosphate salt (chloroquine diphosphate salt, chloroquine), were purchased from Sigma-Aldrich and Roche Applied Sciences (Sant Cugat del Vallés, Barcelona, Spain).

### Cell culture

Caco-2/TC7 cell line was used in this study as previous results have described SERT expression and activity in this cell line [5]. Cells were incubated at 37 °C in a controlled atmosphere of 5% CO_2_ and cultured in high glucose DMEM, supplemented with 2 mM glutamine, 100 U/ml penicillin, 100 μg/ml streptomycin, 1% nonessential amino acids, and 20% heat-inactivated fetal bovine serum (FBS) from Life Technologies (Carlsbad, CA, USA). Medium was renewed two days after cell seeding and daily afterward. All the experiments were carried out 14 days post-seeding accordingly to preliminary results [5]. Twenty-four hours before the experiments, cells were cultured under serum-free conditions. For 5-HT uptake assays, cells were seeded in both 24-well plates and 12-well permeable polyester (PET) membranes at a density of 4 × 10^4^ cells per well. Cells were cultured in 75 cm^2^ flasks with a density of 9 × 10^5^ cells per flask for protein analyses and in 6-well plates with a density of 2 × 10^5^ cells per well for mRNA extractions. ODN and the different chemicals were added to the culture medium at different periods and concentrations as indicated. Preliminary assessment of cell monolayer morphology was achieved in the cell culture after the treatments concluding that none of the different conditions affected morphology, proliferation or monolayer integrity of Caco-2/TC7 cells (data not shown).

### Animals

Male C57BL/10 mice (wild type, WT) and deficient mice for TLR9 (*tlr9*^*−/−*^) were generously provided by Dr. Marina Freudenberg (Max-Planck-Institut für Immunbiologie, Freiburg). Mice were bred at the Centro de Investigación y Tecnología Agroalimentaria (CITA, Zaragoza, Spain) being all genotypes checked periodically. In all the experiments, mice from 10 to 12 weeks of age were maintained under pathogen-free conditions on a 12-h light/dark cycle with food and water ad libitum. Experiments were approved (P136/12) by the Ethic Committee for Animal Experiments of the Zaragoza University accordingly with the Spanish Policy for Animal Protection RD53/2013, which meets the European Convention for the Protection of Vertebrate Animals used for Experimental and other Scientific Purposes (Council of Europe Nº 123, Strasbourg 1985) and the European Union Directive 2010/63/EU on the protection of animals used for scientific purposes. Mice were euthanized by cervical dislocation, and then, intestinal tract (ileum and colon) was removed and washed with a cold NaCl 0.9% immediately after death. RNAlater from Qiagen (Hilden, Germany) was used for collecting RNA tissue samples, and then, samples were frozen at -80 °C. For protein measurements, tissue samples were frozen in ice-cold isopropyl alcohol and then stored at -80 °C.

### 5-HT uptake studies

Uptake measurements were taken in Caco-2/TC7 cells using 24- well plates, either under control condition or after treatment with specific TLR9 agonist. The transport medium composition was: 137 mM NaCl, 4.7 mM KCl, 1.2 mM KH_2_PO_4_, 1.2 mM MgSO_4_, 2.5 mM CaCl_2_, 10 mM HEPES pH 7.4, 4 mM glutamine, 1 mM ascorbic acid, 0.1% BSA, and both 0.2 μM 5-HT and [^3^H]-5-HT (1.5 μCi/ml) as substrate. Before uptake experiment, cells were incubated at 37 °C in an atmosphere of 5% CO_2_ with substrate-free transport medium for 30 min. Then, cells were immediately washed with substrate free-transport medium and then incubated with transport medium at 37 °C for 6 min. Uptake was stopped by removing the transport medium and washing the cells twice with an ice-cold transport medium with 20 μM 5-HT. Later, cells were solubilized in 0.1 N NaOH for radioactivity counting (Wallac Liquid Scintillation Counter, PerkinElmer) and protein measurement by Bradford method from Bio-Rad (Hercules, CA, USA) with BSA as a standard. All results were calculated in pmol 5-HT/mg protein and are expressed as a percentage of control value (100%).

5-HT fluxes were carried out in cells seeded in 12-well permeable polyester (PET) membranes as previously described [5]. These inserts establish apical (A) and basal (B) compartments. Apical to basal (A-B) flux was measured after adding 0.1 μM 5-HT plus [^3^H]-5-HT (2.5 μCi/ml) to apical compartment. Then, samples were taken from basal compartment every 10 min being replaced with fresh medium. These results were calculated in pmol 5-HT/10 min and were expressed as a percentage of the control (100%). Both before and after treatment, cell monolayer integrity and confluence were checked by measuring transepithelial resistance (TER) with an Epithelial Voltohmmeter (Millicell Electrical resistance system, Millipore).

### RNA extraction, reverse transcription and real-time PCR

RNeasy mini kit from Qiagen (Hilden, Germany) was used for extracting total RNA, respectively, of both, cells seeded in 6-well plates and intestinal tissue of mice. Before total RNA extraction, intestinal mice tissue was thawed in an ice-cold RTL buffer (Qiagen) and homogenized using the Ultra Turrax T25 from IKA (Staufen, Germany). Then, intestinal tissue and Caco-2/TC7 lysates were transferred in a QIAshredder column (Qiagen) following the manufacturer’s instructions. The extracted RNA (1 μg) was used as a template for the synthesis of cDNA by using oligo(dT) primers and reverse transcriptase (Lucigen, Middleton, USA). Negative control was carried out without reverse transcriptase. Resulting cDNA by reverse transcription (RT) was used to determine *tlr9, sert, tph1, tph2, 5-ht1a, 5-ht2a, 5-ht2b, 5-ht2c, 5-ht3, 5-ht4, 5-ht7, gapdh and hprt1* mRNA expression in intestinal tract of mice, whereas Caco-2 cells cDNA was used for analyzing *SERT*, *TLR9*, *GAPDH* and *HPRT1* mRNA level. Real-time PCR was carried out by using SYBR green and the specific primers indicated in Table 1. GAPDH and HPRT1 were used as calibrators. All of the samples were determined in triplicate, obtaining the mean Ct from the three runs. Relative mRNA expression under each experimental condition was expressed as ΔCt = Ct_gene_ – Ct_calibrator_. Thus, relative mRNA expression was calculated as ΔΔCt = ΔCt_treatment_ – ΔCt_control_. Finally, levels of relative gene expression were transformed and expressed as fold difference (= 2^−ΔΔCt^).

**Table 1:** Primers designed from human (h) and mice (m) sequences of the different genes quantifies by real-time PCR

Gene	Sense and antisense primers
*hSERT*	5’ GGCCTGGAAGGTGTGATCA 3’ // 5’ GCGCTTGGCCCAGATGT 3’
*hTLR9*	5’ AGTCCTCGACCTGGCAGGAA 3’ // 5’ GCGTTGGCGCTAAGGTTGA 3’
*hHPRT1*	5’ CTGACCTGCTGGATTACA 3’ // 5’ GCGACCTTGACCATCTTT 3’
*hGAPDH*	5’ CATGACCACAGTCCATGCCATCACT 3’ // 5’ TGAGGTCCACCACCCTGTTGCTGTA 3’
*mSert*	5’ GGCAACATCTGGCGTTTTCC 3’ // 5’ ATTTCGGTGGTACTGGCCCA 3’
*mTlr9*	5’ ACTTCGTCCACCTGTCCAA 3’ // 5’ AGGAAGGTTCTGGGCTCAAT 3’
*mTph1*	5’ CACGAGTGCAAGCCAAGGTTT 3’ // 5’ AGTTTCCAGCCCCGACATCAG 3’
*mTph2*	5’ GAGTTGCTCCACGCTTTGC 3’ // 5’ ACACTCAGTCTACATCCATCCC 3’
*m5-htr*_*1a*_	5’ TCTGTGAGAGCAGTTGCCACAT 3’ // 5’ AGCGGCAGAACTTGCACTTGAT 3’
*m5-htr*_*2a*_	5’ TGCCGTCTGGATTTACCTGGATGT 3’ //5’ TACGGATATGGCAGTCCACACCAT 3’
*m5-htr*_*2b*_	5’ AGGAAATGAAGCAGACTGTGGAGG 3’ //5’ CAGTGCAACAGCCAGAATCACAAG 3’
*m5-htr*_*2c*_	5’ ATAGCCGGTTCAATTCGCGGACTA 3’ // 5’ TGCTTTCGTCCCTCAGTCCAATCA 3’
*m5-htr*_*3*_	5’ TCTTGCTGCCCAGTATCTTCCTCA 3’ // 5’ TTATGCACCAGCCGCACAATGAAG 3’
*m5-htr*_*4*_	5’ AATGCAAGGCTGGAACAACATCGG 3’ // 5’ TGTATCTGCTGGGCATGCTCCTTA 3’
*m5-htr*_*7*_	5’ TCTTCGGATGGGCTCAGAATGT 3’ // 5’ AACTTGTGTTTGGCTGCGCT 3’
*mHprt1*	5’ CTGGTGAAAAGGACCTCTCGAA 3’ // 5’ CTGAAGTACTCATTATAGTCAAGGGCAT 3’
*mGapdh*	5’ AACGACCCCTTCATTGAC 3’ // 5’ TCCACGACATACTCAGCAC 3’

### Protein expression of Caco-2/TC7 brush border-enriched fraction and mice intestinal samples

Caco-2/TC7 cells cultured in 75 cm^2^ flasks and mice intestinal samples (ileum and colon) were used for protein expression determination. Cell brush border membrane-enriched fraction from Caco-2/TC7 cells was extracted following the method described in a previous paper [5]. First of all, cells were washed twice with PBS and re-suspended with an ice-cold Tris-mannitol buffer (2 mM Tris, 50 mM mannitol, pH 7.1) with protease inhibitors and 0.02% sodium azide. Then samples were disrupted by Potter–Elvehjem and sonication (fifteen 1-s bursts, 60 W) obtaining cell lysate. Brush border-enriched fraction from cells was obtained by adding 20 mM CaCl_2_ to cell lysates for 10 min and centrifuged for 10 min at 950 g. Finally, the supernatant was centrifuged for 30 min at 40,000 g and the pellet was re-suspended in a phosphate buffer (10 mM KH_2_PO_4_/K_2_HPO_4_ pH 6.8) to obtain the brush border-enriched fraction.

Mice intestinal samples (ileum and colon) were washed and homogenized using ultra-turrax in the same Tris-mannitol buffer pH 7.1 as Caco-2/TC7 cells. Then, samples were disrupted by sonication. Protein content was measured using the Bradford method (Bio-Rad).

Brush border-enriched fraction and cell lysate from Caco-2/TC7 cells, as well as ileum and colon homogenates of wild type (WT) and *tlr9*^*−/−*^ mice, were electrophoresed in 8% SDS-PAGE gels and transferred to PVDF membranes by electroblotting. Plots were blocked with 4% non-fat dried milk plus 1% BSA and probed with a goat polyclonal antibody anti-human and anti-mouse SERT (1:1000) and a mouse monoclonal antibody anti-human and anti-mouse TLR9 (1:1000). Primary antibodies were detected using specific secondary antibodies coupled with horseradish peroxidase with the Western Bright Sirius HRP substrate from Advansta (Menlo Park, CA, USA). Western blot image was obtained by using VersaDoc™ from Imaging System Bio-Rad. After stripping, membranes were re-probed with goat polyclonal anti-human and anti-mouse β-actin as a load control. SERT/β-actin and TLR9/β-actin protein ratio were measured in densitometry units using Quantity One Analysis Software from Bio-Rad. All the results were expressed as a percentage of the control value (100%).

### Statistical analyses

Results are expressed as means ± the standard error of the mean (SEM). The statistical comparisons were made by using Student’s unpaired t-test or one-way ANOVA. One-way ANOVA was followed by the Bonferroni post-test with a confidence interval of 95% (*p* < 0.05). Normal distribution was previously confirmed with the D’Agostino-Pearson test. Every statistical analysis is indicated in each figure. Statistical analysis was performed using Prism GraphPad (Prism version 7.0, GraphPad Software, San Diego, CA). Data are available upon request.

## Results

### TLR9 activation downregulates SERT

**Fig. 1:**
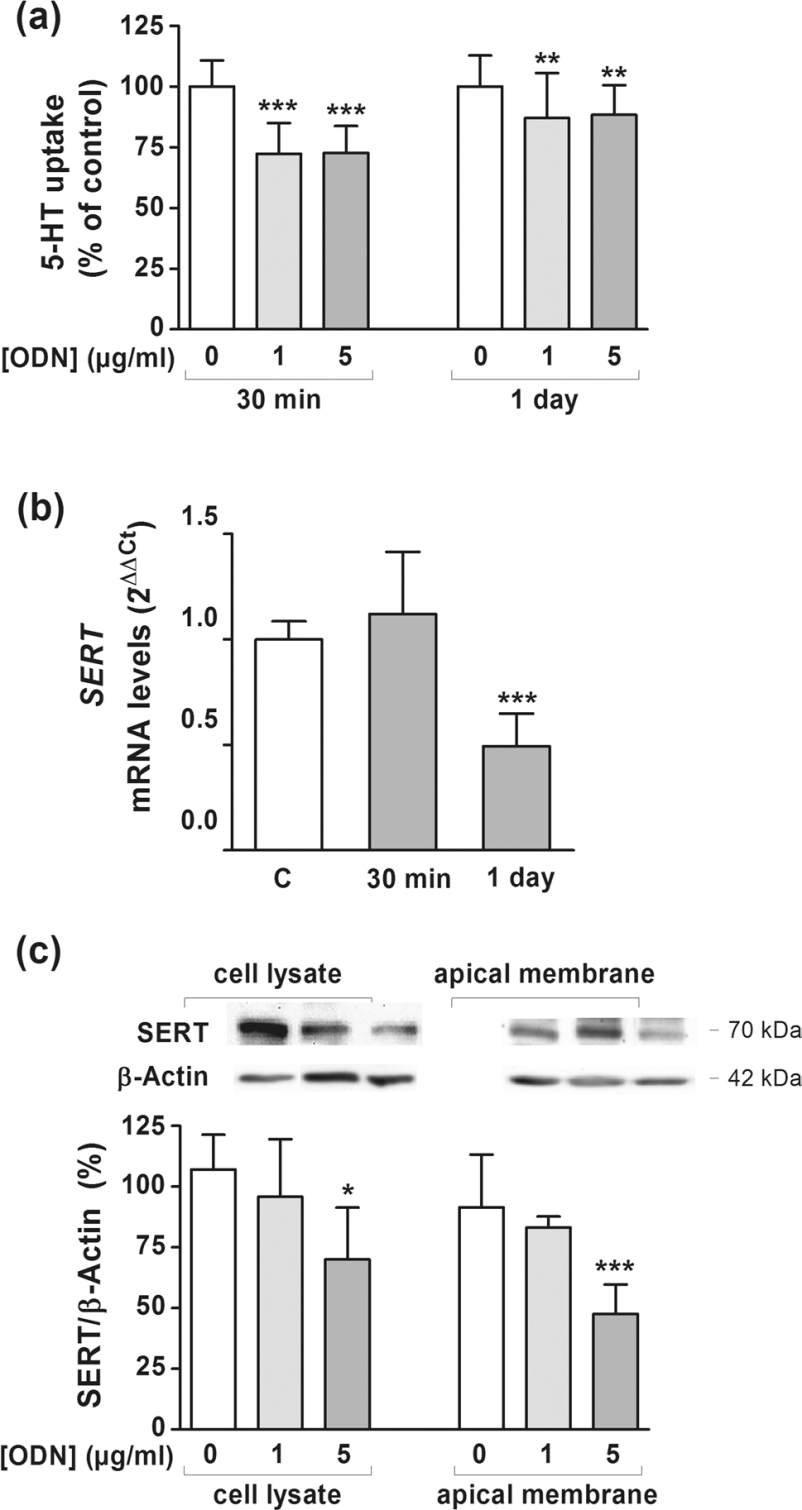
**TLR9 activation by ODN inhibits SERT function and expression. a.** 5-HT uptake was measured after 6-min incubation of 5-HT 0.2 μM. ODN concentrations assayed were 1 and 5 μg/ml. The stimulation periods were 30 min (short-term) and 1 day (long-term). The results are expressed as the percentage of the control (100%) and are the mean plus SD of seven independent experiments. Absolute control values were 7.77 ± 0.74 and 8.72 ± 0.68 pmol 5-HT/mg protein at 30 min and 1 day, respectively. **b.** Real-time PCR analysis of *SERT* mRNA expression level in cells treated for 30 min and 1 day with ODN 5 μg/ml. Relative quantification was performed with the comparative Ct method (2.^–ΔΔCt^) normalized by HPRT1 and GAPDH mean. Results are expressed as arbitrary units (control = 1) and are the mean plus SD of six independent experiments. **c.** Immunodetection and quantification of SERT protein expression by western blot in cell lysate and apical membrane from Caco-2/TC7 cells treated with ODN 1 and 5 μg/ml for 1 day using β-actin as an internal control of the protein load (SERT/β-actin ratio). The results are expressed as percentage of the control value and are the mean plus SD of four independent experiments. *P < 0.05, **P < 0.01 and ***P < 0.001 compared with the control value

TLR9 was activated by ODN (class C CpG oligonucleotide) at 1 or 5 µg/ml for short (30 min) and long (1 day) periods followed by measurement of SERT activity. The data showed that TLR9 activation decreased 5-HT uptake following short- and long-term TLR9 stimulation (Fig. 1a). In order to know the molecular effects of TLR9 activation, *SERT* mRNA and protein levels were measured in Caco-2/TC7 cells treated with ODN. Short-term activation of TLR9 (30 min) did not affect *SERT* mRNA expression, whereas long-term ODN treatment (1 day) significantly decreased *sert* mRNA level (Fig. 1b) at 5 µg/ml. SERT protein expression was also measured after long-term ODN treatment (1 day). The results revealed that SERT protein level was significantly diminished in both cell lysate and apical membrane of treated cells with TLR9 agonist at 5 µg/ml (Fig. 1c).

**Fig. 2:**
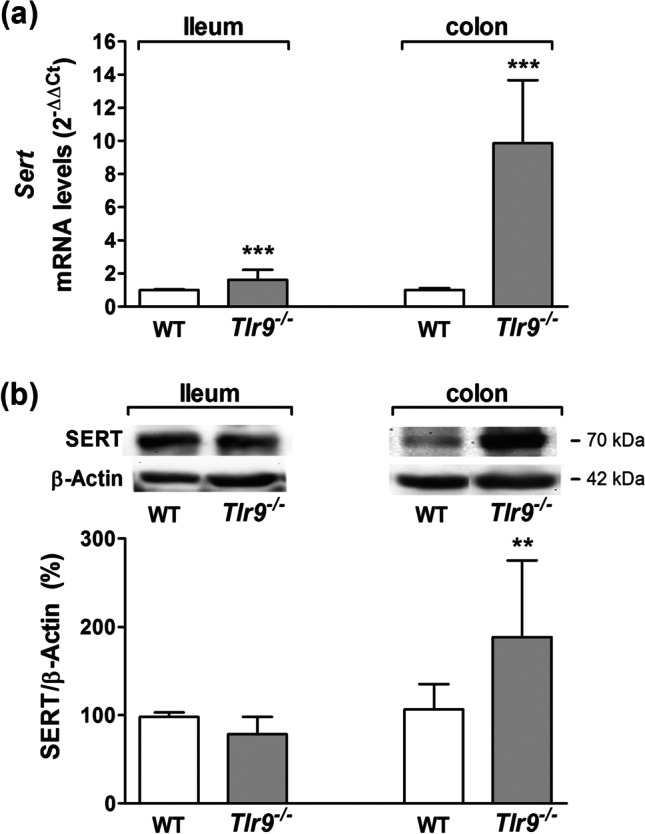
**SERT expression on intestinal tract of Tlr9**^**−/−**^** mice.** *Sert* mRNA and protein expression was measured in ileum and colon from WT (wild type) and *Tlr9*^−/−^ mice. **a**. *Sert* mRNA was measured by real-time PCR using the specific primers shown in Table 1. Relative quantification was performed in triplicate with the comparative Ct method (2.^–ΔΔCt^) normalized by HPRT1 and GAPDH mean. Results are expressed as arbitrary units (control = 1) and are the mean plus SD of five animals. **b**. SERT protein expression was quantified by western blot using β-actin as internal control of the protein load (SERT/β-actin ratio). The results are expressed as percentage of the control value and are the mean plus SD of five animals. **P < 0.01 and ***P < 0.001 compared with the control value

To corroborate these results, we analyzed the SERT expression in intestine of *Tlr9*^*−/−*^ mice. In ileum, *Sert* mRNA expression was increased with respect to WT (Fig. 2a) although SERT protein level was unchanged (Fig. 2b). Meanwhile in colon, *Sert* mRNA expression was strongly increased (Fig. 2a) and consequently, SERT protein level was also higher in colon of *tlr9*^*−/−*^ mice (Fig. 2b).

### Intracellular pathways involved in TLR9 effects on SERT

**Fig. 3:**
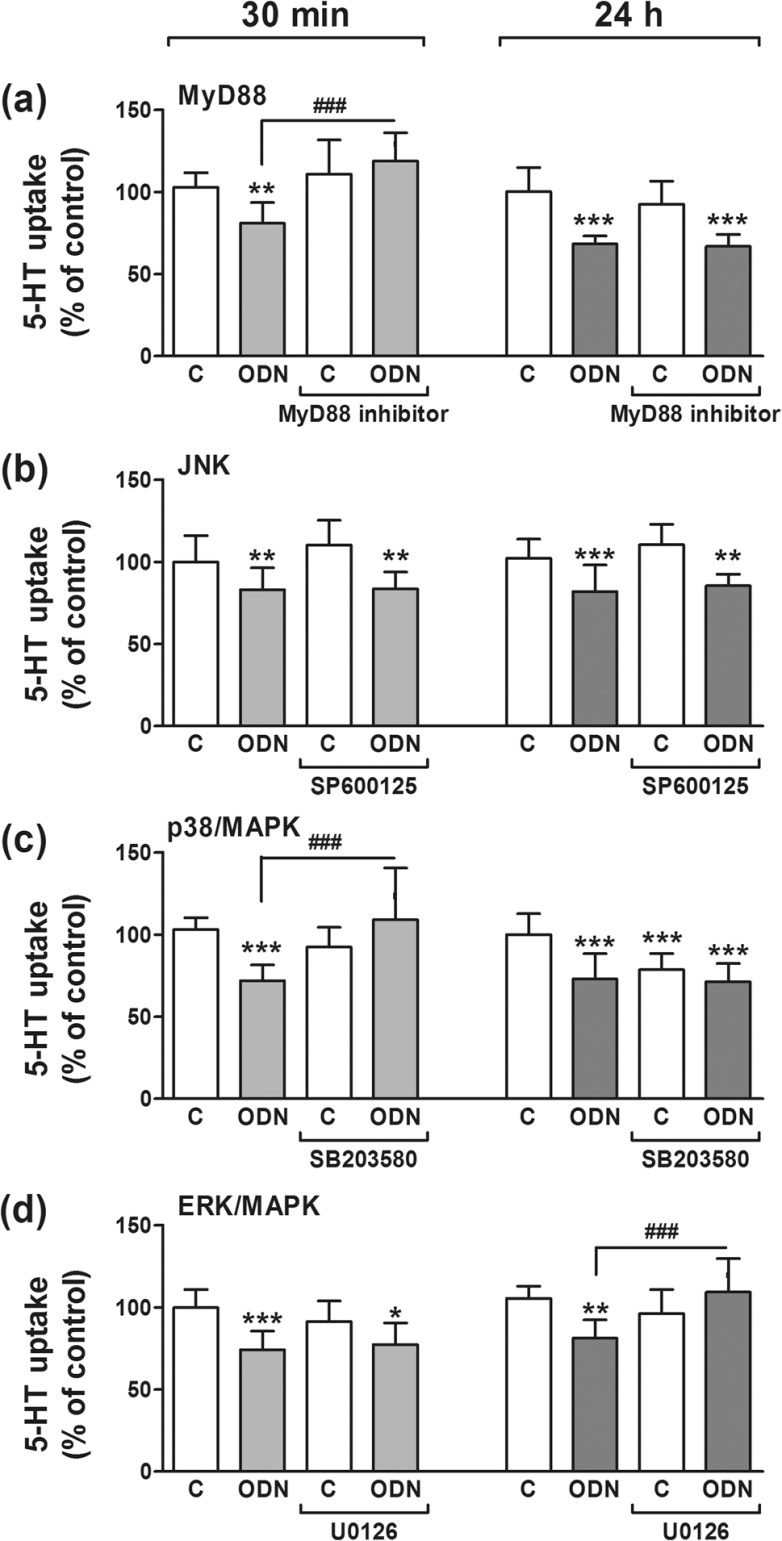
**Analysis of intracellular pathways involved in TLR9 activation on SERT function.** Caco-2/TC7 cells were treated for 30 min and 1 day with ODN 5 μg/ml with or without the pre-treatment with the corresponding inhibitor of the intracellular pathways assayed. Uptake of 5-HT was measured after 6 min of incubation with 5-HT 0.2 μM. The results are expressed as percentage of the control (100%). **a. MyD88 pathway.** Cells were 1 h pre-treated with or without MyD88 inhibitor (Pepinh-MYD; 40 µM). Results are the mean plus SD of five independent experiments. **b. JNK pathway.** Cells were 1 h pre-treated with or without SP600125 1 μM (JNK inhibitor). Results are the mean plus SD of seven independent experiments. **c. p38/MAPK pathway.** Cells were 1 h pre-treated with or without SB203580 1 μM (p38/MAPK inhibitor). Results are the mean plus SD of seven independent experiments. **d. ERK/MAPK pathway.** Cells were 1 h pre-treated with or without U0126 1 μM (ERK/MAPK inhibitor). Results are the mean plus SD of seven independent experiments. *P < 0.05, **P < 0.01 and ***P < 0.001 compared with the control value; ^###^*P* < 0.001 compared with ODN-treated cells

Since TLR9 activation is able to inhibit SERT activity, intracellular signaling pathways involved were studied. Firstly, MyD88 downstream signaling was studied since this is the major effector in TLRs activation [6]. Therefore, we treated cells with TLR9 agonist for 30 min or 1 day (Fig. 3a) with or without a MyD88 specific inhibitor (Pepinh-MYD; 40 µM), 1 h before the treatment with ODN. The results showed that MyD88 inhibition could reverse the short-term ODN effect on 5-HT uptake (Fig. 3a) but not the 5-HT uptake inhibition in the long-term.

Secondly, we studied the JNK intracellular pathway as it has been shown to be involved in the TLR9-mediated inflammatory response [7]. One hour before TLR9 stimulation with ODN, cells were pre-treated with a JNK inhibitor (SP600125; 1 µM) followed by measurement of 5-HT uptake. The results obtained showed that JNK inhibitor in both short-and long-term period of stimulations with ODN did not reverse the inhibitory effect of TLR9 activation on SERT (Fig. 3b).

We next analyzed whether the effect of TLR9 activation on SERT inhibition could be driven by p38/MAPK signaling as it has previously been implicated in several TLR9-dependent processes [8]. Consequently, cells were treated with a p38/MAPK inhibitor (SB203580; 1 µM) 1 h before the short- and long-term treatment with ODN. Inhibition of p38/MAPK reversed the TLR9 short-term effect on SERT in Caco-2/TC7 cells (Fig. 3c); however, this intracellular pathway seemed not to be involved in the long-term TLR9 inhibitory effect on SERT (Fig. 3c).

Following on from the identification of a role for p38/MAPK in TLR9 short-term effects, we analyzed whether the effect of ODN on SERT could be through ERK/MAPK signaling as ERK/MAPK seems to be involved in several TLR9 effects [8]. Thus, cells were pre-treated with an ERK inhibitor (U0126; 1 µM) 1 h before the treatment with ODN for short (30 min) and long term (1 day) stimulations. The results determined that ERK/MAPK downstream signaling is not be involved in the TLR9 short-term effect on SERT, whereas ERK/MAPK inhibitor may reverse the TLR9 inhibitory effect on SERT during long-term treatment with ODN (Fig. 3d).

### Downstream signaling involvement in TLR9 effects on SERT

**Fig. 4:**
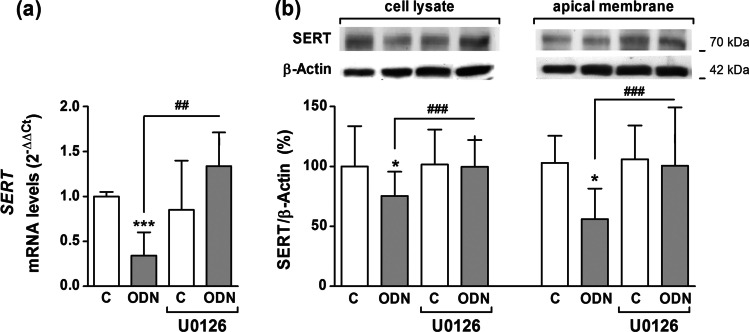
**Role of ERK/MAPK on SERT expression after TLR9 long-term activation.** Caco-2/TC7 cells were treated for 1 day with ODN 5 μg/ml with or without 1-h pre-treatment with U0126 1 μM (ERK/MAPK inhibitor). **a.** Real-time PCR analysis of *SERT* mRNA expression. Relative quantification was performed in triplicate with the comparative Ct method (2.^–ΔΔCt^) normalized by HPRT1 and GAPDH mean. Results are expressed as arbitrary units (control = 1) and are the mean plus SD of four independent experiments. **b.** Immunodetection and quantification of SERT protein expression by western blot in cell lysate and apical membrane from Caco-2/TC7 cells. Results are expressed as SERT/β-actin ratio and are the mean plus SD of four independent experiments. *P < 0.05, **P < 0.01 and ***P < 0.001 compared with the control value; ###*P* < 0.001 compared with ODN-treated cells

For the results shown above, the intracellular pathways of MyD88 and p38/MAPK would seem to be involved in the short-term effects of TLR9 activation. As we have shown previously, SERT expression was unchanged by short-term ODN treatment, and consequently the inhibition of MyD88 or p38/MAPK prior to TLR9 agonist for short stimulation does not modify the expression of SERT either (data not shown). However, results of the present work have shown that ERK/MAPK downstream signaling independent of MyD88 adaptor seems to be implicated in TLR9 long period effect on SERT. We carried out *SERT* mRNA level and protein analysis to know the influence of ERK/MAPK intracellular pathway. The results showed that the decrease in *SERT* mRNA expression induced by ODN was reversed by the inhibition of ERK/MAPK (Fig. 4a). In addition, reduction of SERT protein amounts in both cell lysate and apical membrane after ODN treatment was also reversed with the addition of ERK/MAPK specific inhibitor (Fig. 4b).

### TLR9 in apical membrane is involved on SERT inhibition

Previous studies have described that TLR9 is expressed in both apical and endosomal membrane [9]. Therefore, we aim to determinate which TLR9 localization is responsible for SERT inhibition in Caco-2/TC7 cells.

**Fig. 5:**
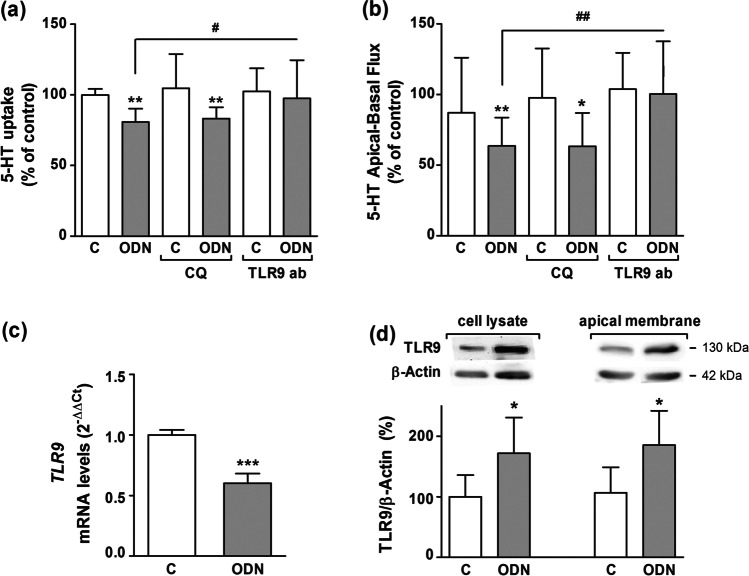
**TLR9 located in cell membrane is involved in SERT inhibition.** Caco-2/TC7 cells were treated for 1 day with ODN 5 μg/ml with or without a 1-h pre-treatment with chloroquine (CQ) 20 μM or TLR9 antibody (TLR9 Ab) 1:100. **a**. Uptake of 5-HT was measured after 6 min of incubation with 5-HT 0.2 μM. The results are expressed as the percentage of the control (100%). Results are the mean plus SD of four independent experiments. Absolute control value was 1.584 ± 0.35 pmol 5-HT/mg protein. **b.** 5-HT apical-to-basal fluxes. 5-HT was used at 0.1 μM, and samples were taken every 10 min from the basal side. The results are expressed as the percentage of the control value (100%) and are the mean plus SD of five independent experiments. Absolute control values in pmol 5 HT/10 min were 0.31 ± 0.04. **c**. Real-time PCR analysis of *TLR9* mRNA expression in Caco-2/TC7 cells treated during 1 day with ODN 5 μg/ml. Relative quantification was performed in triplicate with the comparative Ct method (2^–ΔΔCt^) normalized by HPRT1 and GAPDH mean. Results are expressed as arbitrary units (control = 1) and are the mean plus SD of three independent experiments. **d**. Immunodetection and quantification of TLR9 protein expression by western blot in cell lysate and apical membrane from Caco-2/TC7 cells treated with ODN 5 μg/ml for 1 day. Results are expressed as SERT/β-actin ratio and are the mean plus SD of three independent experiments. *P < 0.05, **P < 0.01 and ***P < 0.001 compared with the control value, unless otherwise indicated; ^#^*P* < 0.05 and.^##^*P* < 0.01 compared with ODN-treated cells

Cells were pre-treated for 1 h prior to stimulation with either chloroquine (CQ; 20 µM), or specific TLR9 antibody (TLR9 ab). CQ is an inhibitor of endosomal acidification that blocks the activity of endosomal components whereas the apical treatment with a specific TLR9 antibody may inhibit the activity of apically expressed TLR9. The results showed that the effect of ODN long-term treatment on 5-HT uptake was not reversed by CQ, meanwhile TLR9 specific antibody inhibits the long-term effect of ODN treatment effect on SERT (Fig. 5a). Therefore, apically expressed TLR9 rather that endosomal TLR9 is responsible for the long-term TLR9-mediated effect on SERT.

In order to clarify these data, apical-basal 5-HT fluxes were performed after both endosomal (CQ; 20 µM) and specific TLR9 antibody treatment. The results of apical-basal 5-HT fluxes seem to support previous data, suggesting that TLR9 long-term effect on SERT would be due to the apical TLR9 activation (Fig. 5b).

As previous results showed that apical TLR9 activation by ODN inhibits SERT, we evaluated if ODN could modify TLR9 expression (mRNA and protein). *TLR9* mRNA expression was reduced after long-term ODN treatment (Fig. 5c). Surprisingly, TLR9 protein level was significantly increased in both cell lysate and apical membrane (Fig. 5d).

### TLR9 expression after 5-HT treatment in Caco-2/TC7 cells

**Fig. 6:**
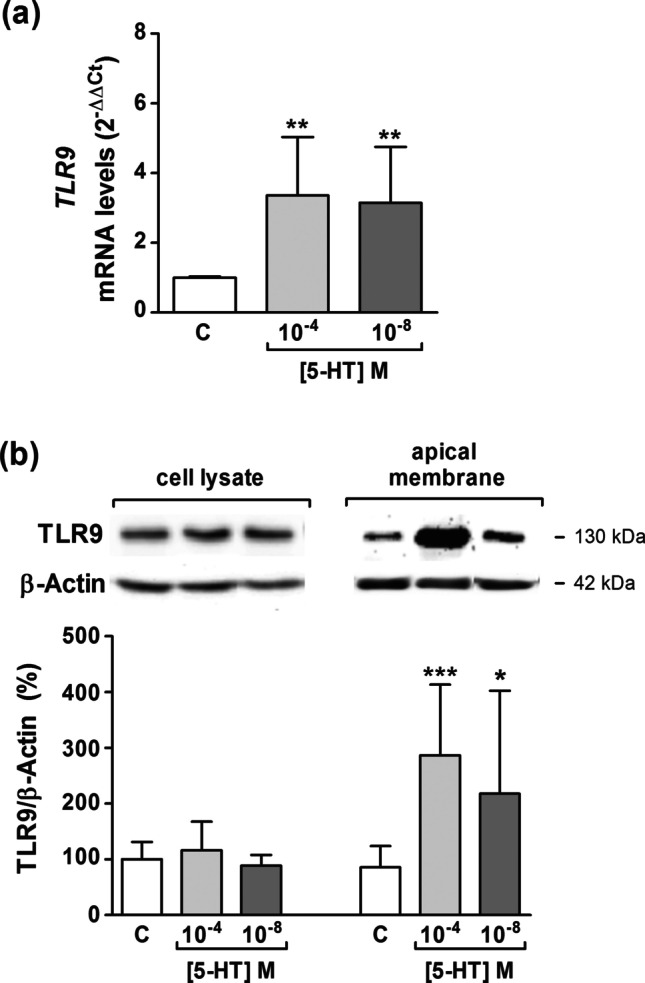
**TLR9 expression is modulated by 5-HT.** Caco-2/TC7 cells were treated for 1 day with 5-HT 10^–4^ and 10^–8^ M **a.** Real-time PCR analysis of *TLR9* mRNA expression. Relative quantification was performed in triplicate with the comparative Ct method (2.^–ΔΔCt^) normalized by HPRT1 and GAPDH mean. Results are expressed as arbitrary units (control = 1) and are the mean plus SD of three independent experiments. **b.** Immunodetection and quantification of TLR9 protein expression by western blot in cell lysate and apical membrane. Results are expressed as TLR9/β-actin ratio and are the mean plus SD of four independent experiments. *P < 0.05, **P < 0.01 and ***P < 0.001 compared with the control value

We have demonstrated in the present study that TLR9 activation decreases both SERT expression and activity, consequently this could induce an increase in the extracellular availability of 5-HT in the intestinal epithelium. To know whether 5-HT may affect TLR9 expression, cells were treated for 1 day with an inflammatory (10^–4^ M) and a physiological (10^–8^ M) 5-HT concentration [1]. Data indicated that 5-HT treatment increased both TLR9 mRNA (Fig. 6a) and protein in the apical membrane (Fig. 6b), indicating a cross-regulation between serotonin and TLR9.

### *Serotonergic system expression in intestinal tract of Tlr9*.^*−/−*^* mice*

Since TLR9 activation downregulates SERT expression and activity in Caco-2/TC7 cells, in turn 5-HT increases TLR9 expression, we decided to verify TLR9 interdependence with intestinal serotonergic system in *ex-vivo* model. Therefore, *Sert, Tph* enzymes (*Tph1* and *Tph2*) and 5-HT receptors (*1A, 2A, 2B, 2C, 3, 4,* and *7*) mRNA expression was measured in ileum and colon of *Tlr9*^*−/−*^ mice.

**Fig. 7:**
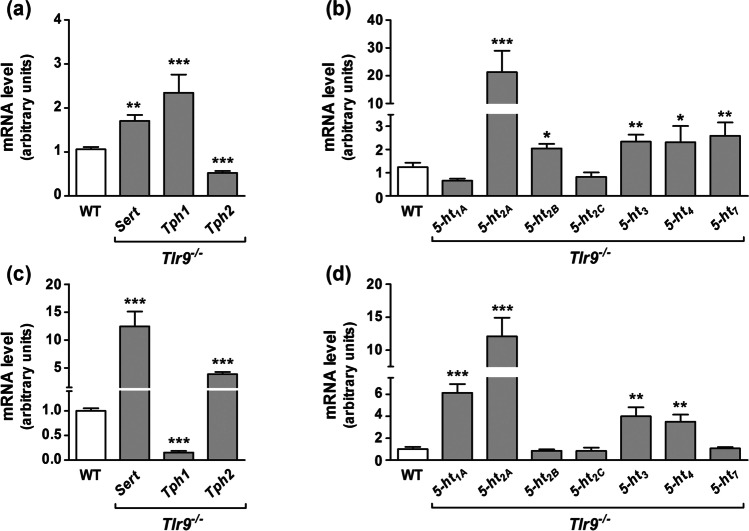
**Gene expression of serotonergic system in ileum and colon of *Tlr9***^**−/−**^** mice.** *Sert*, TPH enzymes (*Tph1* and *Tph2*) **(a, c)** and 5-HT receptors (*1A*, *2A*, *2B*, *2C*, *3*, *4*, and *7*) **(b, d)** mRNA expression was measured in ileum **(a, b)** and colon **(c, d)** of wild type (WT) and *Tlr9*^*−/−*^ mice by real-time PCR. Relative quantification was performed in triplicate with the comparative Ct method (2.^–ΔΔCt^) normalized by HPRT1 and GAPDH mean. Results are expressed as arbitrary units (control = 1) and are the mean plus SD of five animals. *P < 0.05, **P < 0.01 and ***P < 0.001 compared with the control value (WT)

*Sert* mRNA was significantly increased in both the ileum and colon of *Tlr9*^*−/−*^ mice with respect to WT (Fig. 7a and c). Furthermore, the results showed that *tph1* and *tph2* were inversely expressed in *tlr9*^*−/−*^ mice in ileum and colon. *Tph1* was increased in ileum but decreased in colon, whereas *Tph2* was decreased in ileum and increased in colon (Fig. 7a and c). In addition, mRNA expression of the 5-HT receptors *2A, 2B, 3, 4* and *7* was increased in the ileum of *Tlr9*^*−/−*^ mice (Fig. 7b), whereas mRNA expression of the *1A 2A, 3* and *4* 5-HT receptors was higher in the colon of *Tlr9*^*−/−*^ mice when compared to WT mice (Fig. 7d).

## Discussion

TLR9 plays a vital role in intestinal physiology developing either a protective or tolerogenic response to pathogenic and beneficial microorganisms, respectively [10]; and it also seems to be critical in the intestinal repair and enterocyte proliferation [11]. Moreover, several authors have previously reported that microbiota can promote 5-HT synthesis from enterochromaffin cells by increasing TPH1 expression [12] and may also produce 5-HT by itself [13]. Here, we have described for the first time that the innate receptor TLR9 could modulate the intestinal serotonergic system, proving a potential relationship between microbiota and serotonergic system.

Serotonergic system is essential for maintaining intestinal homeostasis; in fact, 5-HT controls numerous gastrointestinal functions. 5-HT levels depends on synthesis in enterochromaffin cells and are regulated mainly by serotonin transporter (SERT) activity. 5-HT is mainly released from basal border of the enterochromaffin cells but also is releases into lumen through the apical membrane. The neighboring enterocytes take up 5-HT by SERT, which is expressed at the apical and basolateral membranes. SERT expression is much higher in the small intestine than in the colon, In the intestinal epithelial cells, SERT is detected in the cytoplasm, being more concentrated in the upper portions of cells, but also was visible in the intracellular compartments, close to the basolateral membrane. Thus, SERT on the tips of the enterocytes makes luminal 5-HT uptake possible while its basolateral expression suggests 5-HT reuptake from the lamina propria [14]. Here, we have observed that TLR9 activation decreases SERT activity. Similar downregulation has been previously demonstrated by activation of other TLRs as TLR2, TLR3, TLR4 or TLR10 [1]. Moreover, TLR9 activation significantly decreases SERT protein level in both cell lysate and apical membrane suggesting a transcriptional or posttranscriptional modification of SERT, which would affect its protein synthesis.

Our results suggest both MyD88-dependent and MyD88-independent TLR9 activation would affect SERT at during both short and long-term stimulations, respectively. We show that short-term effect on SERT would be mediated by MyD88 and its downstream signaling through p38/MAPK, without changes in SERT mRNA and protein levels. In agreement with our results, other studies have also implicated p38/MAPK in SERT inhibition by pattern recognition receptors [5].

Consequently, these results suggest that short-term regulation of SERT by TLR9 would not involve changes in transcriptional or translational mechanisms leading to a fast serotonergic response with the increase of extracellular 5-HT. Extracellular intestinal 5-HT increment could, in turn, evoke the recruitment of pro-inflammatory factors in the gut in order to protect the intestinal epithelium against harmful elements such as pathogens [15]. These findings are in agreement with previous studies which pointed toward the involvement of MyD88 and p38/MAPK in TLR9 inflammatory processes [8].

MyD88 adaptor is a chief component in the intestinal homeostasis mediated by TLRs. Several studies have pointed MyD88 as essential for TLR9 signaling pathway in apoptosis and inflammation [6], where both TLR9 endocytosis and endosomal maturation will be necessary [16]. However, our work shows that TLR9 long-term effect would be mediated by ERK/MAPK independent of MyD88 adaptor, since inhibition of this downstream signaling reversed the long-term TLR9 effect on SERT activity, but also inhibited changes of SERT mRNA and protein expression. This is not the first time that a TLR could act independently of MyD88, since TLR3 and TLR4 activation can trigger MyD88-independent downstream signaling [17]. In fact, TLR9 agonist may play a protective role via MyD88-independent pathway [18] and through a non-inflammatory ERK/MAPK signaling [19]. Moreover, ERK/MAPK intracellular pathway would be related to 5-HT effects [20] and even with 5-HT release from enterochromaffin cells [21]. Therefore, it could be that the protective apical TLR9 role may be due to MyD88-independent downstream signaling, whereas endosomal TLR9 might activate MyD88 pathway leading to a pro-inflammatory responses. Within this context, recent studies have highlighted that apical TLR9 in IECs is absent in germ-free mice meanwhile in conventional animals with beneficial microbiota, TLR9 is active and expressed in the apical surface [22]. Then, our results suggest that long-term TLR9 activation would result in different outcome from the rapid TLR9 activation which suggests a dual role of TLR9 in the gut.

Delving into the TLR9 effect on SERT, our data suggest that SERT inhibition may be mediated by the TLR9 expressed in the cell apical membrane. There have been many studies on TLR9 cellular localization, proving that it is localized in late endosomes or lysosomes mediating the response to unmethylated CpG motifs [4]. Intracellular TLR9 activation requires the acidification of endosomes and lysosomes, which can be inhibited by chloroquine which in-turn inhibits the TLR9 response to CpG [4]. However, a remarkable study showed that TLR9 localized at the cell surface can be activated by CpG despite blocking of endosomal/lysosomal acidification, that would prevent recognition of self-DNA but facilitate access to pathogenic DNA [4]. Moreover, another study reported a different response between apical and basolateral TLR9 activation in intestinal epithelial cells. TLR9 basolateral activation leads to an upregulation of pro-inflammatory IL-8 triggering NF-κB inflammatory pathways, whereas apical activation of TLR9 induced polyubiquitinated IκBα, preventing NF-κB activation. These authors also highlighted that apical TLR9 signal inhibited inflammatory signals from basolateral TLR5 or apical TLR2 activation [23]. Surprisingly, apical TLR9 would not be expressed in germ-free mice meanwhile in conventional mice with normal microbiota show high levels of apical TLR9 [24] supporting the idea that intracellular TLR9 would be involved in immune reaction in the gut meanwhile apical TLR9 would mediate the beneficial bacterial signals of the microbiota with the intestine and highlight the importance of apical TLR9 in microbiota-gut signaling. Then, TLR9 would not only be involved directly in the regulation of the serotonergic system in the gut, but also could indirectly control the intestinal serotonergic system by its interaction with the intestinal microbiota. These data suggest the pivotal role of TLR9 in the intercommunication of the serotonergic system with the intestinal defense and gut microbiota.

In agreement with our results, some studies have already shown that TLR9 is overexpressed in the cell surface after treatment with pathogenic bacterial DNA [22]. Our data shows that ODN treatment in Caco-2/TC7 cells decreased *TLR9* mRNA expression but increased TLR9 protein level in both cell lysate and apical membrane. The lack of correlation between mRNA expression and protein level could be due to the complex and different posttranscriptional mechanisms that are not well defined. Moreover, TLR9 protein could have a different half-live respect to its mRNA due to different period of synthesis and degradation [24]. Therefore, ODN could increase TLR9 protein synthesis and, in a feedback loop, cells could control TLR9 protein over-synthesis by decreasing TLR9 mRNA level [25].

In this study, we have reported that TLR9 may also be influenced by the serotonergic system, since our data indicates that 5-HT increases *TLR9* mRNA level and also protein expression in apical membrane, suggesting a control homeostatic mechanism. Similarly, we have previously shown that 5-HT could increase TLR2, downregulate NOD1 or not modify TLR3 expression [1], so we cannot discard an indirect 5-HT effect on TLR9 expression. Recent investigations have reported that 5-HT produced by microbiota could signal through TLRs to develop responses maintaining intestinal homeostasis [2]. Moreover, 5-HT induces inflammatory responses and seems to be increased in inflammatory diseases acting as a pro-inflammatory factor [26]. In this context, TLR9 could act as a protective innate immune component since recent studies have shown the important protective role of TLR9 in intestinal inflammation, acting as an anti-inflammatory mediator in the intestine [11]. Supporting the beneficial role of TLR9, other authors have indicated that TLR9 activation releases antimicrobial peptides and growth factors from Paneth cells protecting the small intestine [27].

TLR9-deficient mice show a reduced tight junction expression with an increased susceptibility to colonic injury [11], suffering an exaggerated intestinal damage. In accordance with our results from Caco-2/TC7 cells, *Sert* mRNA expression was increased in the intestinal tract of *Tlr9*^*−/−*^ mice. However increased SERT protein was only observed in the colon of *Tlr9*^*−/−*^ mice.

The enzymatic activity of TPH1 and TPH2 are well known to be the rate-limiting step in the synthesis of 5-HT. TPH1 is expressed in enterochromaffin cells, which are located mainly in small intestine, whereas TPH2 is expressed in enteric neurons whose 5-HT produced may affect principally colon motility [28]. Our study on intestinal tract of *Tlr9*^*−/−*^ mice shows that mRNA expression of *Tph1* was increased in ileum and decreased in colon, whereas *Tph2* would be decreased in ileum and increased in colon. As both enzymes are altered in intestinal tract of *Tlr9*^*−/−*^ mice, it seems to imply a deeper implication of TLR9 in the serotonergic system, indicating that TLR9 not only modifies SERT expression and activity, but also influences intestinal 5-HT synthesis in both ileum and colon. In fact, in ileum of *Tlr9*^*−/−*^mice TPH1 level was increased significantly with respect to TPH2 expression, whereas in colon of *Tlr9*^*−/−*^ mice, TPH2 expression was increased with respect to TPH1 level. Although TLR9 appears to be a regulator of TPH1 and TPH2 expression, it has recently been proven that TLR2 would play a pivotal role in gut 5-HT production. A possible interrelation between TLR9 and TLR2 remains unknown.

Serotonergic signaling has multiple and pleitropic effects that are mediated by a wide family of serotonin receptors. Intestinal tract of *Tlr9*^*−/−*^ mice display an altered 5-HT receptors expression. 5-HT receptor 2A, 2B, 3, 4 and 7 mRNA were increased in ileum, whereas in colon an increased mRNA expression of *1A, 2A, 3* and *4* 5-HT receptors was observed.

5-HT1A is an important receptor in intestinal tract [29] playing a chief role in the regulation of 5-HT release [30]. The increase of *5-HT1A* mRNA level in colon of *Tlr9*^*−/−*^ mice agrees with a study where activation of this receptor increased the 5-HT uptake in Caco-2 cells resulting in an increase in SERT activity [29]. 5-HT receptors *2A* and *2B* mRNA expression was also increased in the intestinal tract of *Tlr9*^*−/−*^mice. 5-HT2A and 5-HT2B are involved in several gastrointestinal disorders such as inflammatory bowel syndrome [31]. 5-HT3 receptor has also been reported to be an important regulator of inflammatory and immune responses as its inactivation by specific 5-HT3 antagonist reduced neutrophil infiltration and decreased proinflammatory cytokines in a colitis model [32]. In the present study, *5-HT3* mRNA expression was increased in both the ileum and colon of *Tlr9*^*−/−*^ mice, which could agree with the protective role of TLR9 in intestinal tract. In fact, *Tlr9*^*−/−*^ mice are more susceptible to a dextran sulfate sodium colitis model [11].

Expression of 5-HT receptors 4 and 7, that regulate intestinal motility, was also altered. *5-HT4* mRNA expression is increased in the ileum and colon of *Tlr9*-deficient mice, whereas 5-HT7 mRNA is increased only in the ileum; these changes would drive in an abnormal intestinal motility. Supporting our results, patients with irritable bowel syndrome present an overexpression of TLR9 [33].

Previous studies have shown that mice deficient for TLR2 or TLR4 display alterations of 5-HT receptors, which reinforce a closer relationship between 5-HT receptors and TLRs as we have described here. However, our results show that 5-HT2C receptor would not be modified by deletion of TLR9, but it would be altered in mice deficient for other TLRs. These data hold that every TLR could act in different ways on intestinal serotonergic system, developing diverse responses [1].

In summary, our work shows the strong implication of TLR9 to modulate intestinal serotonergic system. TLR9 activation is able to downregulate intestinal SERT activity and expression in a MyD88-dependent manner during short-term stimulations and in a MyD88-independent way during longer period of TLR9 activation. Moreover, this effect appears to be mediated by p38/MAPK and ERK/MAPK intracellular pathways as a function of time of activation. In return, it has also been demonstrated that 5-HT controls TLR9 expression, in a feedback mechanism of regulation. Finally, we have shown the deep implication of TLR9 in intestinal serotonergic system expression, since the enzymes involved in 5-HT synthesis and 5-HT receptors are also altered in the intestinal tract of *Tlr9*^*−/−*^ mice.

Recent studies have proven that TLR9 has a key role in several pathologies such as gastrointestinal cancer and inflammation, especially in inflammatory bowel diseases (IBD). In fact, TLR9 seems to play a protective role and its activation has been proposed as a target for treatment of IBD [34]. Our study emphasizes the importance of microbial effects via TLRs activation, which in turn regulates intestinal serotonergic system, preserving intestinal homeostasis and preventing intestinal damage. Increased the knowledge about host-microbiota interaction in intestinal tract through both TLRs and serotonergic system can provide new understanding about intestinal homeostasis and inflammation.
